# Molecular Epidemiology, Clinical Manifestations, Decolonization Strategies, and Treatment Options of Methicillin-Susceptible Staphylococcus Aureus Infection in Neonates

**DOI:** 10.3390/ijms262311430

**Published:** 2025-11-26

**Authors:** Aikaterini Nikolaou, Maria Baltogianni, Niki Dermitzaki, Chrysanthi Maria Tsiogka, Nikitas Chatzigiannis, Foteini Balomenou, Vasileios Giapros

**Affiliations:** Neonatal Intensive Care Unit, School of Medicine, University of Ioannina, 45500 Ioannina, Greece; nikaikaterini@gmail.com (A.N.); mbalt@doctors.org.uk (M.B.); n.dermitzaki@uoi.gr (N.D.); chrisatsiogka@gmail.com (C.M.T.); nikihatzi@gmail.com (N.C.); f.balomenou@uoi.gr (F.B.)

**Keywords:** MSSA, neonates, NICU infections, colonisation, decolonisation, β-lactams

## Abstract

Methicillin-susceptible Staphylococcus aureus (MSSA) remains a major pathogen in neonatal intensive care units (NICUs), with colonization and infection posing significant risks. MSSA colonization occurs in up to 42.8% of neonates, while 12–41% of healthcare personnel also carry MSSA, contributing to nosocomial transmission. MSSA accounts for approximately 12% of neonatal *S. aureus* bloodstream infections, with mortality rates up to 20.5%, particularly among very-low-birth-weight infants. This review analyzes the molecular attributes, epidemiology, risk factors, clinical presentations, decontamination methods, and treatment alternatives for MSSA infections in newborns. MSSA strains show considerable genetic heterogeneity, being distinguished by a wide variety of sequence types (STs) and Staphylococcal Protein A types (SpA). They harbor several pathogenicity genes—including hemolysins, superantigens, adhesins, and Panton–Valentine leukocidin (PVL)—which are implicated in severe infections, while biofilm-associated genes enhance environmental persistence. Prematurity, low birth weight, prolonged hospitalization, and exposure to invasive devices are key risk factors. Active surveillance and decolonization programs have achieved reductions of up to 73% in MSSA infections. β-lactam antibiotics remain first-line therapy, with adjunctive aminoglycosides reserved for severe cases. Ongoing genomic surveillance and targeted preventive strategies are essential to reduce MSSA-associated morbidity and mortality in this vulnerable population.

## 1. Introduction

Soon after completing his undergraduate studies at the University of Aberdeen (Scotland), Alexander Ogston first identified *Staphylococcus* in 1880, describing it as a Gram-positive, spherical “micrococcus,” a term derived from the Greek word meaning “bunches of grapes” [1]. In 1961, the United Kingdom reported the first isolates of *Staphylococcus aureus* resistant to methicillin (methicillin-resistant *S. aureus*, MRSA). Subsequently, the term MSSA (methicillin-susceptible *S. aureus*) was introduced to define strains that remain susceptible to methicillin and other β-lactam antibiotics [2]. *S. aureus* is a major human pathogen responsible for both community- and hospital-acquired infections, ranging from mild skin lesions to life-threatening diseases [3]. Although the incidence of invasive *S. aureus* infections has increased over the past two decades—mainly due to MRSA—MSSA infections in hospitalized full-term and preterm neonates (up to 28 days of postnatal age, including preterm neonates who remain hospitalized in the NICU beyond this period) have declined to approximately 23 per 10,000 but remain more frequent than MRSA across all birth-weight categories, exhibiting comparable mortality rates [4].

MSSA has emerged as the predominant strain globally, despite regional variability in distribution [5,6,7,8], and continues to represent a major public health concern [1,9]. In pediatric populations, MSSA accounts for approximately 70–84% of invasive *S. aureus* infections, while MRSA represents 16–30%. Hospital-acquired infections occur in 26% of MRSA cases compared with 13% of MSSA cases [10,11,12]. In neonates, MSSA infections are three to four times more frequent than MRSA infections [13,14]. The incidence of MSSA bacteremia in infants under one year of age is estimated at ~60 cases per 100,000 annually, nearly half (47%) of which occur during the neonatal period [15]. These data indicate that MSSA remains the dominant pathogen, while MRSA, although less frequent, poses a higher risk of hospital-acquired, multidrug-resistant infection. Continuous surveillance, prudent antibiotic use, and strict infection control are therefore essential, particularly in neonatal populations [9,10,11,12,13,14,15,16].

Emerging evidence indicates that genes associated with virulence play a critical role in determining the pathogenic potential and clinical severity of MSSA infections. This effect is further intensified by the spread and persistence of antimicrobial-resistant clones in both hospital and community environments. Key virulence determinants, such as toxins, adhesins, and immune evasion factors, have been linked to severe outcomes, including sepsis, pneumonia, and endocarditis, particularly among neonates and immunocompromised pediatric patients [17]. The genetic heterogeneity of MSSA, coupled with the coexistence of antibiotic resistance genes, enhances strain adaptability, promotes persistence within healthcare settings, and increases the risk of outbreaks [18]. Comprehensive characterization of these virulence-associated genes is essential for informing effective infection control strategies, guiding the development of novel therapeutics, and shaping preventive measures, including potential vaccination approaches, to reduce MSSA-related morbidity and mortality.

This narrative review provides an updated synthesis of current global evidence (up to August 2025) regarding MSSA infections in neonates, integrating recent epidemiological data, molecular analyses, and clinical management strategies. It combines quantitative findings from large-scale surveillance studies with recent genomic insights to provide a comprehensive and up-to-date overview of MSSA burden and control in neonatal intensive care units. The impact of this review lies in its potential to guide neonatal infection prevention policies and antimicrobial stewardship programs by identifying high-risk populations, summarizing effective decolonization interventions, and highlighting molecular mechanisms associated with persistence and virulence. By bridging clinical, microbiological, and genomic perspectives, it contributes to the design of targeted preventive and therapeutic approaches aimed at reducing MSSA-associated morbidity and mortality among neonates.

**Figure 1:**
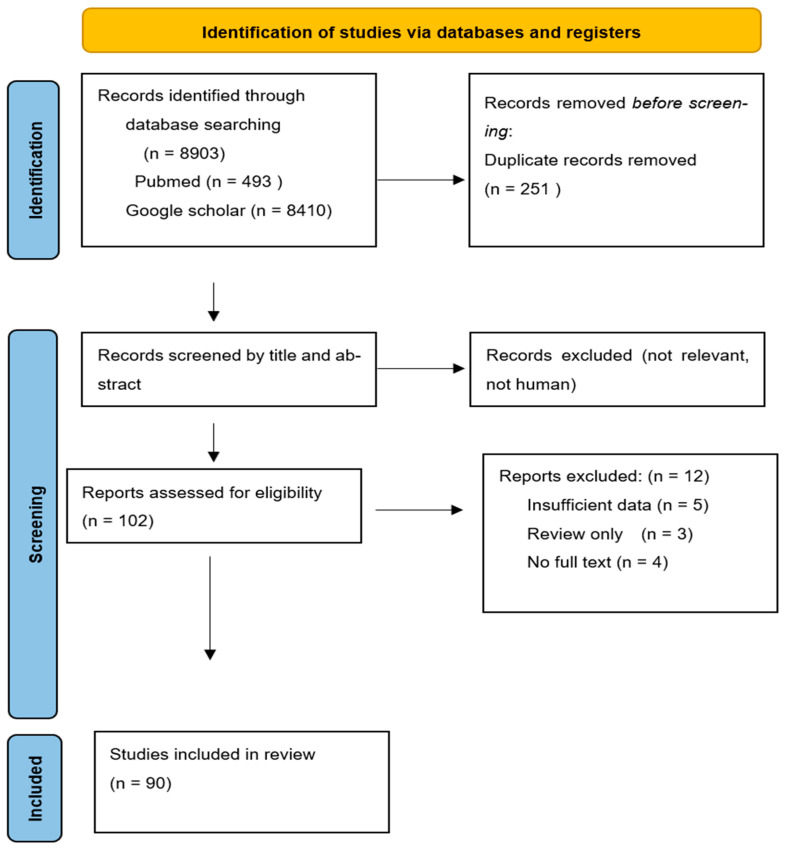
PRISMA flow diagram of search procedure.

A comprehensive literature search was conducted in July and August 2025 to identify studies reporting MSSA infections in neonates, infants, and children, with particular focus on neonatal populations. Two independent researchers searched PubMed and Google Scholar databases using the following terms: (“Methicillin-sensitive *Staphylococcus aureus*” OR MSSA) AND (neonate OR newborn OR infant OR neonatology). Inclusion criteria were as follows: human studies involving neonates (≤28 days), infants (<1 year), or pediatric patients, including both hospitalized and community-acquired infections; articles published in English; and clinical trials, observational studies, surveillance studies, or case series providing data on MSSA epidemiology, virulence factors, or clinical management. Relevant studies were selected based on these predefined inclusion and exclusion criteria, focusing on neonatal MSSA infections, and articles not meeting these criteria (e.g., animal studies or adult-only populations) were excluded. Studies published in the database from inception until August 2025 were considered. After screening titles and abstracts, full texts were reviewed, and eligible studies were included. Any disagreements between the two reviewers were resolved through discussion and consensus. As this is a narrative review, no formal assessment of study quality or risk of bias was performed; this has been acknowledged as a limitation. We retrieved 8903 articles, 90 of which were found to be relevant (Figure 1). Table 1 includes the studies/references related to the neonatal age group and NICU.

## 2. Molecular Epidemiology of MSSA

### 2.1. Molecular Characteristics of MSSA

The molecular characteristics of MSSA provide critical insights into its epidemiology, mechanisms of colonization, and potential evolution toward antibiotic resistance. MSSA exhibits considerable genetic diversity, reflected in a wide array of sequence types (STs) and Staphylococcal Protein A (SpA) types. In contrast, MRSA strains frequently display a limited number of dominant clonal lineages, largely due to the dissemination of epidemic strains. Multilocus sequence typing studies have identified ST398 and ST15 among the most commonly observed sequence types in MSSA, although regional variations are documented [38]. This extensive genetic heterogeneity illustrates the organism’s ability to adapt to multiple ecological niches, including human hosts.

Despite susceptibility to methicillin, MSSA strains frequently harbor genes conferring resistance to other antimicrobial agents. The *blaZ* gene, encoding a β-lactamase, is present in approximately 85–90% of isolates and mediates penicillin resistance, which remains widespread globally [39,40]. Other relevant resistance genes include *tet(K)*, associated with tetracycline efflux [41], and *erm(C)*, which methylates 23S rRNA to confer resistance to macrolides [19]. Such findings indicate that MSSA is a potential reservoir for resistance determinants that could be horizontally transferred to other staphylococcal strains or species.

The pathogenic potential of MSSA is mediated by a combination of virulence factors. Hemolysin genes (*hla*, *hld*, *hlgA*, *hlgB*, *hlgC*) contribute to cytolysis and facilitate tissue invasion [42]. Additionally, enterotoxins and superantigens may play a role in eliciting proinflammatory responses in airway epithelium, thereby amplifying local inflammation during infection [42]. Unlike some pandemic MRSA strains, the Panton–Valentine leukocidin (*pvl*) gene is rarely detected in MSSA, yet this does not diminish its clinical relevance [43]. Other virulence determinants, including microbial surface components recognizing adhesive matrix molecules, toxic shock syndrome toxin-1, and enterotoxins, contribute to disease severity, particularly in neonates, whose immune systems are immature.

Immune evasion is further facilitated by the immune evasion cluster, which includes genes such as *sak* (staphylokinase), *chp* (chemotaxis inhibitory protein), and *scn* (staphylococcal complement inhibitor). These genes inhibit complement activation, neutrophil chemotaxis, and fibrin degradation, thereby promoting persistence and colonization [44].

The interplay between molecular features and the clinical behavior of MSSA is complex. Genetic variability results in strains with differing virulence and antimicrobial susceptibility profiles. In neonates, MSSA colonizes the nasopharyngeal and cutaneous epithelium, exploiting the immature immune system to establish infection [45,46]. MSSA infections in neonates can result in pneumonia, sepsis, or endocarditis, highlighting that MSSA pathogenicity may be comparable to that of MRSA, despite methicillin susceptibility. Additionally, MSSA serves as a potential reservoir for the emergence of novel MRSA clones via horizontal transfer of *mecA* or *mecC* through mobile genetic elements such as plasmids, transposons, and phages [47]. Geographic variation in MSSA clones has been reported, with ST398 more prevalent in Asia and ST15/ST30 more common in North America and Europe [47]. Such heterogeneity necessitates continuous molecular surveillance to detect strains with enhanced transmissibility or virulence.

Overall, MSSA represents a highly adaptable pathogen with multiple virulence factors and mechanisms of antimicrobial resistance beyond methicillin susceptibility. Comprehensive knowledge of its genetic architecture, resistance determinants, and virulence profile is essential for devising effective prevention and control strategies, particularly for high-risk neonatal populations. Further research integrating genomic monitoring, polyclonal analysis, and clinical correlation is required to elucidate the role of MSSA in neonatal infections.

### 2.2. Antimicrobial Resistance

MSSA continues to be clinically significant due to its capacity to cause a spectrum of infections ranging from skin and soft tissue infections to life-threatening systemic diseases, including endocarditis, pneumonia, and bacteremia. While MRSA has historically attracted the majority of clinical attention, MSSA exhibits distinct antimicrobial resistance profiles that are highly relevant for treatment decisions, particularly in neonatal NICUs, where therapeutic options are limited.

Although MSSA is defined as methicillin-susceptible, most isolates (>80–90%) exhibit penicillin resistance due to the presence of *blaZ*, which encodes a β-lactamase capable of hydrolyzing penicillin G and related β-lactams [39,40]. Consequently, empiric and targeted therapy for MSSA infections relies on β-lactams that are resistant to penicillinase, including isoxazolylpenicillins (e.g., oxacillin, nafcillin) and first-generation cephalosporins (e.g., cefazolin).

MSSA frequently carries genes that confer resistance to multiple antimicrobial classes. Methylation of 23S rRNA by *erm* genes (*ermA*, *ermC*, *ermT*) produces the macrolide–lincosamide–streptogramin B resistance phenotype, reducing the effectiveness of erythromycin and clindamycin in neonatal isolates; reported prevalence ranges from 25–78%, depending on the gene and regional clonal distribution [40,48,49]. Tetracycline resistance, mediated by *tetK* (efflux) and *tetM* (ribosomal protection), is present in approximately 10–25% of isolates, limiting the clinical utility of tetracyclines for skin and soft-tissue infections [50]. Fusidic acid resistance— increasingly observed in neonatal populations—results from *fusA* mutations or acquisition of *fusB/fusC*, and affects 5–15% of isolates [40,51].

Resistance to mupirocin, a topical agent widely used for NICU decolonization, arises from *ileS* mutations (low-level resistance) or acquisition of *mupA/mupB* (high-level resistance), occurring in 2–10% of isolates and potentially undermining infection-control strategies [20]. Aminoglycoside resistance, mediated by modifying enzymes encoded by *aac*, *ant*, and *aph*, is reported in 5–20% of neonatal MSSA isolates and may limit the use of synergistic β-lactam–aminoglycoside combinations [52,53].

Although MSSA generally remains susceptible to glycopeptides (vancomycin, teicoplanin) and newer antistaphylococcal agents (linezolid, daptomycin), rare cases of vancomycin-intermediate MSSA highlight that even “susceptible” strains can develop adaptive mechanisms under antibiotic pressure. The presence of the agrI quorum-sensing gene in ~20% of isolates has also been linked to increased biofilm formation and persistence in healthcare environments, further complicating eradication efforts [54].

Collectively, these data provide an overview of the main antimicrobial resistance genes in MSSA, illustrating their mechanisms and relevance to neonatal treatment decisions and emphasizing the need for ongoing surveillance, molecular characterization, and tailored antimicrobial stewardship to prevent treatment failure and reduce morbidity in this vulnerable population. (Table 2)

## 3. Epidemiology of MSSA Burden

The epidemiology of *S. aureus*, particularly MSSA, in NICUs remains a critical research focus due to the heightened vulnerability of neonates. MSSA continues to be a leading cause of both hospital- and community-acquired infections in neonatal settings, even in regions where MRSA prevalence has declined following intensive infection-control efforts [21].

Epidemiological data consistently show that MSSA infections are three to four times more common than MRSA infections in neonatal populations, including in healthcare settings with historically high MRSA burdens [22]. For example, weekly surveillance in one NICU identified MSSA colonization in 42.8% of neonates (164/383), with 2.9% (11/383) developing invasive disease—illustrating the substantial colonization burden and associated infection risk [14]. Similarly, a large multicenter cohort reported *S. aureus* colonization in ~22.9% of neonates (predominantly MSSA) and an infection incidence of 1.7% among 590 infants, with colonization strongly predicting subsequent infection (OR ≈ 8.2) [16].

Mortality associated with MSSA infection in neonates is comparable to that seen with MRSA, estimated at ~10–20% in some cohorts [4,14]. A large retrospective study of 468,201 hospitalized infants (postnatal age ≥ 4 days) reported an invasive *S. aureus* incidence of 37.6 per 10,000 infants and an absolute mortality difference of 5.3% (95% CI, 3.8–6.8%) compared with uninfected infants. Very-low-birth-weight infants (<1500 g) were disproportionately affected, accounting for 76.5% of infections and 90.4% of attributable deaths [23]. These findings emphasize that methicillin susceptibility does not necessarily confer lower clinical severity, as MSSA retains multiple virulence determinants and can cause severe neonatal disease.

Geographical and temporal trends in MSSA epidemiology are noteworthy. In Europe and North America, strengthened infection-prevention measures—such as hand hygiene, active surveillance cultures, and decolonization protocols—have reduced MRSA incidence. However, the overall burden of *S. aureus* infections has remained relatively stable due to the persistent prevalence of MSSA. Data from the European Centre for Disease Prevention and Control show that invasive MRSA infections declined from 5.63 per 100,000 population in 2019 to 4.64 per 100,000 in 2023, a ~17.6% reduction [21]. In contrast, the incidence of *S. aureus* infections in hospitalized neonates has remained stable, with 44.8 infections per 10,000 hospitalized patients reported in a multicenter U.S. study [4]. Similarly, in the United States, the Centers for Disease Control and Prevention reports a 16% reduction in hospital-onset MRSA bacteremia as of 2022 in acute-care hospitals, following widespread implementation of hand hygiene, contact precautions, active surveillance, and decolonization strategies [55]. In several Asian regions, MSSA remains a major neonatal pathogen, contributing not only to skin and soft-tissue infections but also to neonatal sepsis. For instance, a study from New Zealand found that 88% of invasive *S. aureus* infections in neonates were caused by MSSA, while MRSA prevalence remained low but stable [56].

The risk of MSSA infection is highest among premature and low-birth-weight infants, who often require prolonged hospitalization and invasive devices (e.g., central venous catheters, mechanical ventilation). Surveillance data indicate that extremely low gestational age (<28 weeks) and use of invasive ventilation are strongly associated with MSSA colonization and subsequent infection [14]. In these vulnerable populations, MSSA can cause severe clinical outcomes comparable to those associated with MRSA [4].

Overall, MSSA remains a pathogen of significant clinical and epidemiological importance in NICUs—similar to MRSA in prevalence, virulence, and transmission potential. Continuous surveillance, targeted decolonization, strict hygiene practices, and improved understanding of regional patterns and transmission pathways are essential to reducing neonatal morbidity and mortality attributable to MSSA.

## 4. MSSA Colonization in Neonates, Clinical Manifestations, and Approaches to Decolonization

### 4.1. MSSA Colonization

Epidemiological studies indicate that *S. aureus* colonizes 20–30% of the general population, with most carriers harboring MSSA [57]. Nasal MSSA carriage among healthy adults typically ranges from 15–30%, though prevalence varies substantially by region [58,59,60]. In NICUs, colonization is also common: in one Chinese cohort, *S. aureus* colonization occurred in 17% of neonates, with 12% carrying MSSA (64/536) [54], while another cohort reported MSSA colonization in 42.8% of neonates (164/383) [21].

Healthcare personnel represent an additional reservoir. One study found that 41.0% of staff (144/351) were colonized with *S. aureus*, but only 2.5% (25/351) carried MRSA, indicating that the majority of carriage was MSSA. Similarly, among German emergency medical service workers, MSSA carriage reached 47.5%, compared with regional surveillance estimates of ~21.9–27.2% in the general population [61,62].

These findings underscore the substantial variability in MSSA carriage across populations and regions, highlighting the importance of local epidemiology when assessing colonization risk and potential transmission in both community and healthcare settings [57,58,59,60,61,62].

Nineteen clinical isolates of PVL-positive, methicillin-susceptible *S. aureus* (PVL-MSSA) were collected from Nottingham University Hospitals NHS Trust. Isolates were selected based on clinical suspicion of PVL-associated disease or characteristic antibiotic resistance patterns, and PVL gene presence was confirmed by the national Staphylococcus Reference Unit [63]. Epidemiological assessment identified small clusters among related patients, but most isolates originated from unrelated neonates and infants and represented a broad range of clinical presentations and specimen types.

Molecular analysis showed that most PVL-MSSA isolates carried a limited repertoire of toxin genes. The enterotoxin genes *seg* and *sei* were detected in nearly 90% of isolates, while classical virulence determinants such as *seb* and *tst* were largely absent [63,64,65,66,67]. Antibiotic susceptibility testing demonstrated high resistance rates to trimethoprim (89.5%) and gentamicin (57.9%), whereas all isolates remained susceptible to clindamycin, rifampin, linezolid, vancomycin, fusidic acid, and teicoplanin, indicating several therapeutic options for neonatal care [63].

Genotyping identified five clonal complexes (CC1, CC22, CC30, CC88, and CC152) and six sequence types (ST22, ST88, ST30, ST1, ST772, and ST1518), with ST22 being the most prevalent (47.4%) [67]. This lineage showed consistent resistance to both trimethoprim and gentamicin. PVL gene analysis revealed seven single-nucleotide polymorphisms relative to the proposed *lukSF-PV* progenitor ΦSLT, with most isolates carrying the H variant [63,68,69,70]. Multiple PVL-encoding bacteriophages were identified; ST30 isolates exhibited the greatest phage diversity (ΦPVL and Φ108PVL), whereas ST22 largely carried a single phage type, suggesting a correlation between clonal lineage and phage content [70].

These findings indicate that PVL-MSSA strains in neonatal and pediatric populations share considerable genetic similarity with PVL-MRSA clones, including ST22, ST88, ST30, and ST1 [71,72,73], supporting the hypothesis of shared evolutionary origins [74,75]. The presence of multiple PVL phage types within certain lineages, such as ST30, suggests repeated phage acquisition events and highlights the genetic diversity and adaptability of PVL-MSSA [68,76]. Clinically, most PVL-MSSA infections manifested as skin and soft-tissue infections, consistent with prior pediatric reports [63].

To summarize these molecular and clinical characteristics, a neonatal-focused table was compiled (Table 3), highlighting sequence type (ST), clonal complex (CC), PVL haplotype, phage type, and key clinical features. This format enables concise comparison across isolates and emphasizes patterns relevant to neonatal disease. For example, ST22 was the predominant lineage and was consistently associated with the H2 PVL haplotype and ΦPVL phage type, while ST30 isolates showed greater phage diversity, suggesting repeated acquisition events. Clinically, most isolates were linked to skin and soft-tissue infections, whereas the sole R haplotype corresponded to a fatal case of necrotizing pneumonia—underscoring the clinical relevance of PVL isoform variation in neonates.

### 4.2. Clinical Manifestations

MSSA infections are a major contributor to morbidity and mortality in NICUs [16,24]. Although research has historically focused on MRSA, MSSA accounts for the majority of clinical infections in neonates, presenting a spectrum from mild skin and soft tissue infections (SSTIs) to severe, life-threatening conditions. This is particularly relevant in the neonatal population, where immature immunity and frequent invasive procedures increase susceptibility [25].

SSTIs—including pustules, abscesses, and cellulitis—represent the most common clinical manifestation in neonates. These infections often serve as the primary portal for bacteremia, facilitated by factors such as immature skin, invasive devices (e.g., intravenous catheters, urinary catheters), and close contact with healthcare personnel and caregivers [3,25].

Bacteremia is the most severe manifestation of MSSA infection in neonates, accounting for a significant proportion of NICU sepsis cases, with mortality rates ranging from 10% to 20% in some cohorts [4,14,26]. A whole-genome sequencing analysis of neonates with MSSA bacteremia (112/926 positive cultures) reported a mortality rate of 20.5% in affected patients [27]. It may occur secondary to SSTI or pneumonia and is strongly associated with central venous catheter use and prolonged hospitalization. While complications such as endocarditis or thrombophlebitis are rare, they have been documented even in neonatal populations.

MSSA pneumonia is less frequent in neonates but can progress rapidly, particularly when PVL-positive strains are involved. Necrotizing pneumonia, though uncommon, is characterized by rapid lung parenchyma destruction, severe respiratory compromise, and high mortality [63].

Other manifestations, such as osteomyelitis and septic arthritis, are more typical in older infants, but in neonates, they usually follow hematogenous spread after bacteremia. Early recognition remains challenging, and diagnostic delays can result in long-term sequelae, including deformities or chronic inflammation.

Rare toxin-mediated syndromes such as toxic shock syndrome (TSS) or staphylococcal enterotoxin–mediated disease can occur even in neonates, illustrating the potent virulence potential of MSSA strains.

Several host and pathogen factors influence disease severity. Low birth weight and prematurity significantly increase susceptibility due to immature immunity and frequent invasive interventions. MSSA colonization, particularly in the nasopharynx, has been linked to increased risk of recurrent infection during prolonged hospitalization [28]. Importantly, the clinical severity of MSSA infections in neonates can be comparable to that of MRSA, reflecting the diverse repertoire of virulence factors rather than antibiotic resistance per se [26].

In conclusion, MSSA infections in neonates range from mild SSTIs to severe sepsis and necrotizing pneumonia. Early detection, careful monitoring, and timely treatment are crucial, particularly in the context of PVL-positive strains, which may cause rapid and severe disease even in previously healthy neonates.

### 4.3. Risk Factors for MSSA Colonization in Neonates

MSSA infections in neonates are not random but linked to defined host- and hospital-related risk factors. Understanding these determinants is crucial for prevention, early detection, and timely treatment.

#### 4.3.1. Host-Related Risk Factors

*Prematurity and low birth weight:*

Preterm neonates, especially those with very low birth weight, face a markedly increased risk of infection. Their immature immune systems and underdeveloped skin and mucosal barriers limit their ability to mount effective defenses against microbial invasion [29]. Multicenter studies show that low-birth-weight infants have a two- to three-fold higher risk of *S. aureus* sepsis, regardless of methicillin susceptibility [26].

*Underlying medical conditions:*

Neonates with congenital heart disease, respiratory distress syndromes, or other chronic health conditions are more prone to infection. Immunosuppressive therapies or immune deficiencies further heighten vulnerability.

*Maternal colonization or infection:*

Vertical transmission of MSSA from colonized or infected mothers has been reported, especially in cases of preterm delivery and cesarean section [30].

#### 4.3.2. Hospital and Environmental Risk Factors

*Invasive devices:*

Central venous catheters, endotracheal tubes, urinary catheters, and monitoring devices provide potential entry points for MSSA. The pathogen can form biofilms on the surfaces of medical devices, enhancing persistence and antimicrobial resistance [31,32]. The presence of a central venous catheter has been identified as one of the strongest predictors of MSSA sepsis in neonates [33].

*Prolonged NICU stay:*

Extended hospitalization increases exposure to colonized individuals and contaminated surfaces. The longer the stay, the greater the likelihood of infection, especially when invasive procedures are required. Studies indicate that the risk of MSSA infection peaks during the first week of life, particularly among preterm neonates [25].

*Colonization and transmission:*

Colonization of the skin or nasopharynx often precedes infection. Transmission may occur vertically from the mother or horizontally through healthcare personnel and NICU surfaces. In a Japanese study, MSSA strains isolated from neonates were genetically identical to those from staff members, confirming the role of nosocomial transmission [28].

*Antibiotic exposure:*

Broad-spectrum antibiotic use, especially cephalosporins and macrolides, can alter the neonatal microbiome and promote MSSA overgrowth [77]. Excessive antibiotic use may also complicate management by selecting for resistant strains.

### 4.4. Precautions Against MSSA Colonization

Neonatal colonization with MSSA represents the first and most critical step in the sequence that can lead to invasive infection. Because most infections in NICUs originate from endogenous colonization or cross-transmission, preventive strategies focus on interrupting both the establishment of colonization and its subsequent spread.

Hand hygiene remains the cornerstone of infection prevention. Numerous studies have demonstrated that the consistent use of alcohol-based hand rubs markedly decreases the transmission of both MSSA and MRSA within NICUs [78]. In addition, strict adherence to aseptic techniques during invasive procedures—such as catheter insertion or ventilator management—significantly reduces the risk of pathogen introduction.

The hospital environment can act as a reservoir for transmission. MSSA has been detected on surfaces, medical equipment, and bed linens. Regular cleaning with antiseptic agents and the use of advanced disinfection technologies, including ultraviolet light and hydrogen peroxide vapor, have been shown to lower the environmental bacterial load [79].

Active surveillance for colonization, through regular sampling of the nasopharynx, skin, and other high-risk sites, is another key component of infection control. Many NICUs implement weekly or biweekly screening cultures. Identification of carriers—whether neonates, healthcare personnel, or caregivers—enables targeted decolonization interventions [33].

Cohorting and isolation of colonized or infected neonates can substantially reduce cross-transmission. Although this strategy may require significant resources, evidence suggests that it effectively lowers the incidence of MSSA colonization in high-risk populations [80].

Topical decolonization therapies, such as intranasal mupirocin and chlorhexidine-based antiseptic washes, have been evaluated in multicenter studies, which reported a 30–50% reduction in new MSSA and MRSA infections in NICUs [81]. Nevertheless, such interventions should be implemented judiciously, as mupirocin resistance has been documented [20]. Table 4 summarizes key measures used to prevent MSSA colonization in neonatal intensive care units [34].

### 4.5. Decolonization Strategies for MSSA in Neonates

Understanding the risk factors that facilitate MSSA colonization has guided the development of decolonization strategies focused on minimizing transmission, reducing bacterial load, and preventing invasive infections among neonates.

Neonatal colonization by MSSA often precedes invasive infection and plays a key role in the epidemiology of *S. aureus* disease in NICUs. Consequently, decolonization strategies are designed to interrupt this process and reduce infection-related morbidity and mortality.

Mupirocin, a topical antibiotic, is commonly employed to eradicate MSSA from the nasopharynx—the primary reservoir for *S. aureus* colonization. Randomized studies have demonstrated that targeted mupirocin administration in colonized neonates significantly decreases the risk of subsequent infection [81]. Its use among colonized healthcare workers has also contributed to limiting nosocomial transmission [20]. However, the emergence of mupirocin-resistant strains restricts long-term or universal application, highlighting the need for judicious use and resistance monitoring.

Chlorhexidine-based skin antisepsis has been shown to reduce microbial burden on the skin surface. In studies involving preterm neonates, routine bathing with chlorhexidine solution correlated with lower MSSA infection rates, without significant adverse events [33]. Nevertheless, caution is warranted when applying chlorhexidine to extremely preterm or very low birth weight neonates, as their skin barrier is immature and may be more susceptible to irritation or systemic absorption.

Evidence suggests that combination decolonization regimens are more effective than monotherapy. Multicenter studies have reported that the combined use of intranasal mupirocin and chlorhexidine baths resulted in up to a 50% reduction in both MSSA and MRSA infections in NICUs [81]. This combined approach is considered among the most validated interventions, but continuous monitoring for potential resistance development remains essential.

Decolonization measures can also extend to parents and healthcare workers, as these groups often serve as reservoirs for MSSA transmission. Studies indicate that a substantial proportion of neonatal infections originate from colonized caregivers [75]. Targeted decolonization of these individuals with mupirocin and chlorhexidine has been associated with interruption of the transmission chain.

There is an ongoing debate regarding targeted versus universal decolonization approaches. Selective strategies—directed only at colonized individuals—minimize the risk of antimicrobial resistance, whereas universal strategies can achieve rapid reductions in colonization but may promote resistance [82]. Most NICUs adopt a balanced approach, integrating routine surveillance cultures with targeted decolonization protocols.

Beyond conventional approaches, emerging research explores the potential of novel interventions, including probiotics, bacteriophages, and antimicrobial peptides, to prevent or reduce MSSA colonization. Although evidence remains limited, preliminary findings suggest that probiotics may support the restoration of normal microbiota and reduce *S. aureus* colonization rates [35].

Continuous monitoring and evaluation are essential to maintain the effectiveness of decolonization programs. Regular screening cultures, antimicrobial resistance surveillance, and staff compliance audits are necessary components of sustainable infection control practices. During routine MSSA surveillance and decolonization in a Level IV NICU, invasive MSSA bloodstream infection rates dropped from 0.37 per 1000 hospital days to 0.00 after intervention, representing an 82% reduction, which demonstrates the crucial importance of adhering to decolonization measures [36].

## 5. Antibiotic Treatment of MSSA Infections in Neonates

While decolonization and infection-control measures play a critical preventive role, timely and appropriate antibiotic therapy remains essential once infection occurs. Evidence regarding optimal antibiotic choice in neonates with MSSA infection is limited, and current recommendations largely derive from broader pediatric and neonatal sepsis data.

Selecting appropriate antibiotic therapy for MSSA infections in neonates is critical, as this population is at increased risk for severe complications. Although MSSA remains susceptible to β-lactam antibiotics, clinical experience indicates that the severity of infection can be comparable to that of methicillin-resistant strains, emphasizing the need for prompt and effective therapy.

Isoxazolylpenicillins, such as oxacillin and nafcillin, are considered first-line agents for treating MSSA infections due to their rapid bactericidal activity and established efficacy [3]. These antibiotics are recommended for serious infections, including pneumonia, sepsis, osteomyelitis, and septic arthritis. Dosage adjustments should be made according to gestational age, postnatal age, weight, and renal function.

Cefazolin, a first-generation cephalosporin, is an effective and generally well-tolerated alternative for MSSA infections, particularly for skin and soft tissue infections or mild cases of sepsis. Some treatment protocols prefer cefazolin over oxacillin because of its lower hepatotoxicity and ease of administration [83].

Aminoglycosides, such as gentamicin, are often added to β-lactam antibiotics during initial empirical therapy, particularly in severe infections or when sepsis is suspected. This combination provides a synergistic effect and accelerates bacterial clearance [52]. However, due to potential nephrotoxicity and ototoxicity, aminoglycosides should be used for a limited duration (typically ≤5 days) before continuing with β-lactam monotherapy.

Vancomycin is not considered a first-line treatment for MSSA. Evidence shows that β-lactam therapy results in superior clinical outcomes compared to vancomycin when MSSA is confirmed [84]. Vancomycin should be reserved for neonates with documented β-lactam allergy or mixed infections involving MRSA. Linezolid may serve as an alternative in cases of multiple drug resistance or intolerance to first-line agents, though its use in neonates is limited, and data remain scarce.

Clindamycin is occasionally used for skin and soft tissue infections, given its excellent tissue penetration and ability to inhibit toxin production. In some regions, fusidic acid is used as adjunctive therapy; however, evidence of its safety and efficacy in neonates is limited [77].

The duration of antibiotic therapy varies depending on infection type and severity. For uncomplicated bacteremia, a minimum course of 10–14 days is generally recommended, while complex infections such as endocarditis or osteomyelitis may require 4–6 weeks of treatment [85]. These recommendations, however, are largely extrapolated from pediatric and general neonatal sepsis management guidelines, as robust, high-quality neonatal MSSA-specific trial data are lacking. Therefore, treatment duration and regimen selection should be individualized, guided by the patient’s clinical response and microbiological findings.

In addition to targeted antimicrobial therapy, supportive management is essential. This includes removal of potentially contaminated catheters or invasive devices, ensuring adequate nutritional support, and, in select cases, the use of immunoglobulin therapy to enhance host defense [37].

Despite the availability of effective antibiotic options, managing MSSA infections in neonates remains challenging due to limited pharmacokinetic data, variable antimicrobial susceptibility patterns, and the emergence of resistance to aminoglycosides and clindamycin. Future directions include the evaluation of novel antistaphylococcal agents, neonatal-specific dosing studies, and the integration of immunotherapeutic or adjunctive strategies into clinical practice.

## 6. Discussion

The reviewed literature emphasizes that MSSA remains a clinically significant pathogen in NICUs, yet its impact is often underappreciated compared to MRSA. Although global research has largely focused on MRSA, multiple studies indicate that MSSA infections occur more frequently and are clinically comparable in severity [26,28]. This observation suggests that current infection surveillance and prevention strategies may under-recognize MSSA, potentially leading to an underestimation of its burden.

Molecular analyses reveal that MSSA strains harbor diverse virulence determinants, including hemolysins, toxins, and biofilm-forming capabilities. Otto [86] demonstrated that *S. aureus* pathogenicity extends beyond methicillin resistance and is closely linked to exotoxin and superantigen production, supporting the notion that MSSA can be highly virulent. Notably, PVL-positive MSSA strains have been associated with necrotizing pneumonia in neonates [63], reinforcing that methicillin susceptibility does not equate to reduced clinical severity. While MSSA isolates remain sensitive to β-lactam antibiotics, resistance to other classes, such as aminoglycosides and clindamycin, has been increasingly reported [52,77], highlighting the need for careful antibiotic stewardship. Turner et al. [77] also emphasize that resistance mechanisms beyond the SCCmec element are emerging, which has practical implications for empirical therapy and resistance surveillance.

Epidemiologically, large multicenter studies report that MSSA infections occur three to four times more frequently than MRSA in NICUs in the United States and China [22,33], whereas certain Asian regions, including parts of Hong Kong, continue to demonstrate higher MRSA prevalence [38]. These geographic differences likely reflect a combination of local antimicrobial policies, infection control practices, and population demographics. Critically, while MRSA incidence has decreased in Europe and North America due to enhanced infection prevention—including rigorous hand hygiene, surveillance cultures, and decolonization protocols—overall *S. aureus* infection rates have remained relatively stable [33,81]. This suggests that MSSA continues to sustain a significant burden despite improvements in MRSA control, highlighting a gap in targeted surveillance for methicillin-susceptible strains.

Colonization is a key precursor to invasive infection. Wertheim et al. [87] identified nasal colonization as a major predictor of *S. aureus* infection, while Toyama et al. [28] confirmed genetic similarity between MSSA isolates from neonates and healthcare workers, underscoring nosocomial transmission pathways. Consistently reported risk factors include prematurity, very low birth weight, prolonged hospitalization, and exposure to invasive devices [25,29], with central venous catheter presence recognized as the strongest predictor of MSSA sepsis [33]. Maternal and neonatal colonization further suggests that preventive interventions may need to extend to caregivers [30]. These findings emphasize that infection prevention must be multifaceted, targeting both patient and environmental reservoirs.

Clinically, MSSA infections range from skin and soft tissue infections to bacteremia and severe pneumonia. Bacteremia is the most clinically significant manifestation, with mortality rates comparable to MRSA [26]. Although necrotizing MSSA pneumonia is rare, its association with PVL-positive clones represents a particularly severe clinical entity [63]. Together, these data demonstrate that methicillin susceptibility is not predictive of a milder clinical course, reinforcing the importance of early recognition and appropriate therapy.

Preventive measures, particularly decolonization using mupirocin and chlorhexidine, have demonstrated measurable reductions in MSSA infection rates in NICUs, with reductions of up to 50–82% reported following implementation [36,81]. However, the potential for mupirocin resistance [20] limits widespread or prolonged use, indicating that targeted, surveillance-guided approaches are preferred [82]. This nuanced understanding highlights that while infection control measures are effective, they must be carefully implemented and continuously monitored to maintain long-term efficacy.

Regarding treatment, β-lactam antibiotics remain the preferred agents for confirmed MSSA infections [3]. Chang et al. [84] reported improved outcomes in MSSA bacteremia with β-lactams compared with vancomycin, while cefazolin has been shown to be an effective and well-tolerated alternative [83]. Aminoglycosides may be used briefly during empirical therapy [52], but their nephrotoxicity necessitates cautious, time-limited use. Importantly, most treatment recommendations for neonatal MSSA are extrapolated from pediatric or adult data, underscoring the need for high-quality, neonatal-specific clinical trials to inform evidence-based management.

## 7. Limitations

This review has several limitations. First, its narrative design may introduce selection bias, as the included studies were not systematically appraised for methodological quality or risk of bias. The selection of articles was based on predefined inclusion and exclusion criteria. Although two independent reviewers screened and selected the studies, the process did not involve a formal quality assessment tool. This limitation has been acknowledged and should be considered when interpreting the findings.

Second, substantial heterogeneity exists across study populations, diagnostic criteria, and reported outcomes, particularly in relation to neonatal-specific MSSA infections. Third, many of the available studies lack detailed methodological descriptions and long-term follow-up, limiting the interpretation of outcomes such as recurrence, resistance emergence, or late morbidity.

Moreover, most available evidence focuses on MRSA, leaving MSSA comparatively underrepresented—especially within neonatal intensive care settings—thereby restricting the generalizability of current findings. Finally, recommendations regarding antibiotic selection, treatment duration, and decolonization practices are largely extrapolated from pediatric or general neonatal sepsis data, rather than being supported by high-quality neonatal MSSA-specific trials. This underscores the need for standardized study designs and robust neonatal-focused research to strengthen the evidence base in this field.

## 8. Future Perspectives

Future research should aim to address the current evidence gaps by implementing multicenter, prospective, and standardized surveillance studies specifically focused on neonatal MSSA infections. Such efforts should include detailed molecular characterization of virulence and resistance determinants, evaluation of treatment protocols and decolonization strategies (e.g., mupirocin, chlorhexidine, and alternative approaches), and rigorous assessment of novel preventive interventions, such as probiotics or innovative disinfectants.

Additionally, monitoring antimicrobial resistance trends and documenting long-term clinical outcomes are crucial to inform evidence-based recommendations. Establishing neonatal MSSA registries would facilitate accurate tracking of incidence, strain distribution, therapeutic outcomes, and emerging resistance, ultimately guiding targeted interventions, improving infection control policies, and reducing morbidity and mortality in this highly vulnerable population.

## 9. Conclusions

In summary, MSSA remains a predominant pathogen in neonatal intensive care units, causing infections ranging from mild skin lesions to severe sepsis and necrotizing pneumonia. Epidemiological evidence indicates that MSSA is more frequent than MRSA in neonates, with ratios of 3:1 to 4:1, and is associated with considerable morbidity and mortality. Major risk factors include prematurity, low birth weight, prolonged hospitalization, and use of invasive devices, while maternal colonization contributes to neonatal colonization. Molecular analyses reveal that MSSA strains carry virulence determinants, such as hemolysins, toxins, and biofilm-forming genes, which enhance pathogenic potential and persistence in healthcare environments. Although β-lactams, particularly cefazolin, remain the treatment of choice, comprehensive infection control measures, including targeted decolonization, hygiene, and environmental interventions, are essential. Given the limited high-quality neonatal-specific data, coordinated surveillance, standardized preventive strategies, and careful antimicrobial stewardship are urgently needed to safeguard this highly vulnerable population and reduce the burden of MSSA infections.

## Figures and Tables

**Table 1:** Neonatal and NICU-Focused MSSA Studies.

Reference	Authors & Year	Age Group/Population	Study Type	NICU Focus	Pathogen	Key Findings/Notes
[4]	Ericson et al., 2015	Hospitalized infants	Clinical/Burden study	Yes	MSSA & MRSA	High incidence in VLBW infants; NICU burden
[13]	Akers et al., 2020	Preterm neonate	Case study	Yes	MSSA	Persistent MSSA bacteremia cleared with cefazolin + ertapenem
[14]	Mahieu et al., 2024	Neonates	Surveillance	Yes	MSSA	MSSA colonization and infection in NICU
[16]	Nurjadi et al., 2021	Neonates	Surveillance	Yes	MSSA	MSSA colonization, transmission, and infection in NICU
[19]	Benito et al., 2015	Healthy neonates	Microbial study	Possible	S. aureus	Characterization of S. aureus from neonatal feces; potential mother-to-infant transmission through breastfeeding
[20]	Patel et al., 2009	Neonates	Surveillance	Yes	MRSA	Mupirocin resistance among MRSA in NICU
[21]	CDC	Neonates	Guidelines	Yes	MSSA & MRSA	Prevention & control of S. aureus infections in NICU
[22]	Geng et al., 2020	Neonates	Epidemiology	Yes	MSSA	MSSA prevalence on NICU admission in China
[23]	Jennings et al., 2025	Hospitalized infants	Clinical/Mortality	Yes	MSSA	Invasive MSSA infection epidemiology and mortality
[24]	O’Reilly et al., 2019	NICU	Retrospective study	Yes	MSSA	Morbidity & mortality of S. aureus bacteremia in NICU
[25]	Vergnano et al., 2011	NICU, England	Epidemiology	Yes	MSSA & MRSA	Neonatal infections surveillance
[26]	Benjamin et al., 2004	Neonates, Preterm	Epidemiology	Yes	MSSA & MRSA	Mortality following blood culture in preterm infants
[27]	Slingerland et al., 2020	Neonates	Whole genome sequencing	Yes	MSSA	Explored nosocomial transmission and virulence of MSSA bacteremia in NICU; WGS revealed potential transmission pathways and virulence factors
[28]	Toyama et al., 2022	NICU	Molecular epidemiology	Yes	MSSA	MSSA molecular epidemiology in NICU
[29]	Stoll et al., 2015	Extremely preterm	Observational study	Yes	MSSA & MRSA	Trends in care, morbidity, and mortality in NICU (1993–2012)
[30]	Jimenez-Truque et al., 2012	Mother-infant pairs	Epidemiology	Yes	MSSA & MRSA	Maternal-infant S. aureus colonization relationship
[31]	Kosmeri et al., 2024	Neonates & Pediatrics	Review	Yes	MSSA & MRSA	Antibiofilm strategies in neonatal/pediatric infections
[32]	Baltogianni et al., 2023	NICU	Review	Yes	MSSA & MRSA	Antibiotic resistance & biofilm management strategies
[33]	Milstone et al., 2013	Critically ill children	Clinical trial	Yes	MRSA	Daily chlorhexidine bathing reduces bacteremia
[34]	Rallis et al., 2023	NICU	Review	Yes	MSSA & MRSA	Antimicrobial resistance and rational antibiotic use in neonatal sepsis
[35]	Patel & Denning, 2015	Neonates	Review	Yes	MSSA & MRSA	Intestinal microbiota and necrotizing enterocolitis
[36]	Balamohan et al., 2018	Neonates	Clinical/Surveillance	Yes	MSSA	Decreased MSSA incidence after implementation of routine surveillance and decolonization; highlights importance of decolonization measures in NICU
[37]	Leibovitz, 2009	Neonates	Review	Yes	MSSA & MRSA	Neonatal sepsis and meningitis: treatment & prophylaxis

AMR—Antimicrobial Resistance; CDC—Centers for Disease Control and Prevention; CHG—Chlorhexidine Gluconate; MSSA—Methicillin-Sensitive *Staphylococcus aureus*; MRSA—Methicillin-Resistant *Staphylococcus aureus*; NEC—Necrotizing Enterocolitis; NICU—Neonatal Intensive Care Unit; VLBW—Very Low Birth Weight; WGS—Whole Genome Sequencing.

**Table 2:** Resistance according to genes.

Gene	Mechanism/Function	Clinical Relevance	Frequency/% Isolates	Reference
*blaZ/blaI/blaR*	Encodes β-lactamase → hydrolyzes penicillin G and other natural β-lactams	Widespread resistance to penicillin; limits β-lactam use	>80–90%	Becker et al., 2014; Boswihi et al., 2024 [39,40]
*erm(A), erm(C)*	23S rRNA methylation → MLSB phenotype (resistance to macrolides, lincosamides, streptogramin B)	Reduced efficacy of clindamycin and erythromycin in neonatal isolates	25–45% (varies by region)	Boswihi et al., 2024; Leclercq et al., 2002 [40,48]
*erm(T)*	23S rRNA methylation → MLSB phenotype	Increased erythromycin/clindamycin resistance in MSSA, including CC398	37–78%	El Mammery et al., 2023 [49]
*tet(K), tet(M)*	Efflux pump (tetK) or ribosomal protection protein (tetM) → tetracycline resistance	Limits the use of tetracyclines in SSTIs	10–25%	Roberts et al., 2005 [50]
*fusB, fusC, fusA mutations*	Protection/modification of EF-G → fusidic acid resistance	Clinically relevant resistance in areas with extensive topical fusidic acid use	5–15%	Boswihi et al., 2024; O’Neill & Chopra, 2006 [40,51]
*mupA, mupB, ileS mutations*	Modification of isoleucyl-tRNA synthetase → mupirocin resistance	Reduces effectiveness of mupirocin-based decolonization strategies, esp. NICU clones (ST1898)	2–10%	Patel et al., 2009 [20]
*aac(6′)-aph(2″), ant, aph*	Aminoglycoside-modifying enzymes → resistance to gentamicin and other aminoglycosides	Limits the synergistic use with β-lactams	5–20%	Chandrakanth et al., 2008; Mahoro et al., 2024 [52,53]
*agrI*	Quorum-sensing gene affecting virulence and biofilm regulation	Correlates with β-lactam resistance and persistence in healthcare environments	~20%	Li et al., 2024 [54]

aac(6′)-aph(2″), ant, aph—aminoglycoside-modifying enzyme genes; agrI—quorum sensing gene affecting virulence/biofilm; blaZ/blaI/blaR—β-lactamase genes; erm(A), erm(C), erm(T)—23S rRNA methylation genes (MLSB resistance); fusA, fusB, fusC—EF-G modification/protection genes (fusidic acid resistance); ileS/mupA/mupB—mupirocin resistance genes; tet(K), tet(M)—tetracycline resistance genes; MLSB—macrolides, lincosamides, streptogramin B; SSTIs—skin and soft tissue infections; NICU—neonatal intensive care unit.

**Table 3:** Molecular and clinical characteristics of PVL-positive MSSA isolates from neonatal patients.

Isolate	Patient/Clinical Context	ST	CC	PVL Haplotype	PVL Phage Type	Antibiotic Resistance	Key Notes
TS6	Neonate, hospital ward cluster	ST22	CC22	H2	ΦPVL	TMP, GEN	Epidemiologically linked to TS9
TS9	Neonate, same ward as TS6	ST22	CC22	H2	ΦPVL	TMP, GEN	Linked cluster
TS12	Neonate, skin infection	ST30	CC30	H1	ΦPVL/Φ108PVL	TMP, GEN, OX, ERY	Outlier H1 PVL SNP, 4-drug resistance
TS17	Relative of TS18/24 patient	ST1	CC1	H2	ΦSa2USA	TMP	Differs at spa locus from TS18/24
TS18	Neonate, patient A	ST30	CC30	H2	ΦPVL/Φ108PVL	TMP, GEN	Similar to TS24
TS24	Neonate, same patient as TS18	ST30	CC30	H2	ΦPVL/Φ108PVL	TMP, GEN	Congruent with TS18
TS21	Neonate, unknown epidemiology	ST88	CC88	H2	Undetermined (morphology only)	TMP	Phage only morphologically identified
TS25	Neonate, skin/soft tissue infection	ST22	CC22	H2	Multiple phages	TMP, GEN	Contains multiple distinct PVL phages

TMP = trimethoprim resistance, GEN = gentamicin resistance, OX = oxacillin resistance, ERY = erythromycin resistance.

**Table 4:** Measures to prevent MSSA colonization.

Bacterial presence in the throat and nasal passages	Positioning the newborn on the mother's breast immediately after birth. Precolonization of the typical α- and/or Á-Streptococcus by applying the mother’s breast milk over and inside the mouths of extremely-low-birth-weight infants immediately upon their admission to the NICU.
Skin microbial flora	Immediate skin-to-skin contact between the mother and newborn should occur in the delivery room right after birth, irrespective of how the delivery took place.
Hand hygiene	Rigorous hand cleanliness before and after caring for newborns.
Gloves	The rate of MSSA isolation decreases when gloves are used for infection control.
Prevent overcrowding	Prevent overcrowding by cohorting and isolating MSSA-positive neonates, implementing barrier precautions, training healthcare staff, and steering clear of congested wards.

MSSA, methicillin-susceptible *Staphylococcus aureus*; NICU, neonatal intensive care unit. Adapted from [34].

## Data Availability

No new data were created or analyzed in this study. Data sharing is not applicable to this article.

## Abbreviations

The following abbreviations are used in this manuscript:

aac(6′)-aph(2″), ant, aph	Aminoglycoside-modifying enzyme genes
agrI	Quorum-sensing gene regulating biofilm formation and virulence
blaI/blaR/blaZ	Genes encoding β-lactamase
CC	Clonal Complex
chp	Chemotaxis inhibitory protein
EF-G	Elongation Factor G
erm(A), erm(C), erm(T)	Genes encoding 23S rRNA methylation
ERY	Erythromycin
fusA, fusB, fusC	Genes/mutations conferring fusidic acid resistance
GEN	Gentamicin
H/R variant	PVL haplotypes
IEC	Immune Evasion Cluster
MLSB	Macrolides, Lincosamides, Streptogramin B
mupA, mupB, ileS	Genes/mutations conferring mupirocin resistance
MSSA	Methicillin-Susceptible *Staphylococcus aureus*
MRSA	Methicillin-Resistant *Staphylococcus aureus*
NICU	Neonatal Intensive Care Unit
OX	Oxacillin
OR	Odds Ratio
pvl/PVL	Panton–Valentine Leukocidin
S. aureus	*Staphylococcus aureus*
sak	Staphylokinase
scn	Staphylococcal complement inhibitor
SCCmec	Staphylococcal Cassette Chromosome mec
SpA	Staphylococcal Protein A
SSTIs	Skin and Soft Tissue Infections
ST	Sequence Type
tet(K), tet(M)	Genes encoding tetracycline resistance
TMP	Trimethoprim
VLBW	Very Low Birth Weight
β-lactam	Beta-lactam
